# Exploring the role of positive direct experience in the adoption of energy efficient technologies: evidence from a Swiss field study on the promotion of low-flow showerheads

**DOI:** 10.1371/journal.pone.0230255

**Published:** 2020-03-16

**Authors:** Uros Tomic, Corinne Moser, Yann Blumer, Michael Stauffacher, Roman Seidl

**Affiliations:** 1 Institute of Sustainable Development, ZHAW School of Engineering, ZHAW, Winterthur, Switzerland; 2 Sustainability Research Group, University of Basel, Basel, Switzerland; 3 econcept AG, Zurich, Switzerland; 4 Institute of Innovation & Entrepreneurship, ZHAW School of Management and Law, ZHAW, Winterthur, Switzerland; 5 Institute for Environmental Decisions (IED), Transdisciplinarity Laboratory (TdLab), ETH Zurich, Zurich, Switzerland; 6 Institute of Radioecology and Radiation Protection, Leibniz University of Hannover, Hannover, Germany; Institute for Advanced Sustainability Studies, GERMANY

## Abstract

Despite the considerable potential of low-flow showerheads to reduce household energy demand, their widespread implementation is still far from being realised. In this study, we compare the joint effect of a contextually embedded intervention in a public swimming pool to promote low-flow showerheads coupled with a mass campaign by a Swiss city’s utility to the stand-alone effect of the mass campaign. We also explore the factors that influence the outcome of the contextually embedded intervention. The quasi-experimental design of the study was possible due to the co-occurring installation of low-flow showerheads in a local public swimming pool and a campaign of a local utility, which offered low-flow showerheads for domestic use at a substantially reduced price. Our findings showed that the combined intervention was substantially more effective than the mass campaign alone. However, this result has to be interpreted with caution owing to the imperfect comparability of the two campaigns. Based on a survey of 402 swimming pool visitors, the study findings demonstrate the crucial role of a positive direct experience in the promotion of low-flow showerheads. This had a significant positive impact on attitudes towards low-flow showerheads, which in turn was found to be the most important determinant of purchase intention. The results suggest that more active communication of energy efficiency measures in public facilities might contribute to reductions in household energy use. Such campaigns can be used to share experiences of energy efficiency technologies and, therefore, promote the use of similar systems at home.

## 1 Introduction

Global society is increasingly adopting sustainable development as an appropriate means to address significant economic, societal and environmental challenges. Based on UNDP Sustainable Development Goal No. 7 regarding “Clean and Affordable Energy”, a target has been set to double the rate of improvement in energy efficiency by 2030 [1]. As the residential sector accounts for around 30% of total energy consumption worldwide [2], implementing programmes to promote the adoption of energy efficiency technologies in private households will play a crucial role in reaching this target.

Research on the energy efficiency gap has already demonstrated that the technologies needed to achieve the global energy efficiency goal are available [3–10]. However, little attention has yet been paid to technologies that reduce warm water consumption. This is surprising because on the aggregate EU level, water heating accounts for 12% of total energy consumption by private households, second only to spatial heating at 68% [11]. Of this 12%, showering accounts for the majority of household warm water consumption [12].

By switching to an efficient low-flow showerhead (LFSH), energy reductions of up to 38% (compared to conventional technology) can be achieved even after taking account of 15% “wasted savings” due to increased shower times in response to the perceived decline in comfort related to the lower flow rate [13]. While the perceived decline in comfort has been identified as a significant barrier to faster adoption of LFSHs [14–17], LFSHs are usually easy to install or remove and do not require specialist equipment or skill. Their installation is, therefore unhindered by a landlord-tenant-dilemma that represents a barrier to the implementation of many energy efficiency measures on the household level. While there have been only a few studies on the prevalence of LFSHs, these suggest that despite their considerable potential, only about half the households in OECD countries possess water flow restrictor taps or LFSHs [14,18].

Against this background, our quasi-experimental study addresses the issue of LFSH adoption through an intervention involving direct experience of an LFSH. This study was conducted during a campaign promoting LFSHs in the city of Winterthur in Switzerland and examined whether faster adoption of LFSHs could be achieved by offering the chance to experience them prior to purchase. This opportunity was provided by a recent refurbishment of a public swimming pool that had been equipped with LFSHs.

## 2 Background

### 2.1 What influences decisions to purchase LFSHs in a specific context?

The theory of planned behaviour (TBP) [19] is considered a highly useful framework for analysing environmentally relevant behaviour—including purchase decisions—in specific contexts [20–28]. The TPB postulates that behavioural achievement can be accurately predicted by behavioural intention and perceived behavioural control. Behavioural intention is defined as “motivational factors that influence behaviour” [19, p. 181] and perceived behavioural control as “perception of the ease or difficulty of performing the behaviour of interest” [19, p. 183]. The TPB also postulates that the behavioural intention itself is determined by perceived behavioural control as well as attitudes and the subjective norm towards the behaviour. Attitudes are defined as “the degree to which a person has a favourable or unfavourable evaluation or appraisal of the behaviour in question” [19, p. 188] and subjective norm as “perceived social pressure to perform or not to perform the behaviour”s [19, p. 188].

Energy-efficient technologies, such as LFSHs, can be promoted through behavioural interventions [26,29]. A large body of literature has evaluated different intervention approaches, such as feedback [30,31], information [32], goal setting [33] and social influence [34–36]. To promote energy efficient technologies, increasing attention has been directed towards social influence approaches, including social norms [34]. This is captured within the TPB framework by the term “subjective norm”. Forgas and Williams [37] define social influence as situations where an individual’s thoughts, feelings or actions are influenced by other people. Thereby, social influence can occur through social interactions, observations and information dissemination [38]. However, an accelerated diffusion of energy efficiency technologies through social influence approaches is more likely to occur in the case of highly visible technologies—such as solar panels [39] or electric cars [40,41]—than less visible ones, such as LFSHs in a domestic setting. This is also in line with Rogers [42], who highlights the importance of innovation visibility in the innovation diffusion process.

Increased visibility could, therefore, be an important factor in the adoption of new technologies. From this perspective, conducting a promotional campaign in a place where showers can be seen (e.g. a gym, sports centre or public swimming pool) presents exciting opportunities. Such places can offer a direct group experience with an LFSH as well as multiple platforms for social interaction to promote LFSHs. From the diffusion of innovation perspective [42], social confirmation by one’s peers could be especially useful in the persuasion stage and in cases of uncertainty regarding any technological benefit. In addition, providing an arena for social interaction between potential adopters with different levels of openness toward innovation—especially early adopters and the early majority—can considerably accelerate the innovation diffusion process.

### 2.2 The importance of direct experience for attitude formation

Direct experience has been shown to influence attitudes substantially, and these are important predictors of behavioural intention, including purchase intention, according to the TPB [19]. It has been recognised that attitudes formed through direct experiences are stronger predictors of subsequent behaviour than indirectly formed attitudes [43–46].

From a marketing perspective, Kempf and Smith [47] and Hoch and Deighton [48] argue that consumers are more responsive to direct rather than virtual experiences for several reasons. Firstly, direct experience is perceived as more vivid, informative and impressive. Secondly, direct experience attracts more attention and is more successful in promoting engagement. Thirdly, information acquired by direct experience is seen as more credible and trustworthy. Hoch [49] also suggests that customers perceive direct experience to be more “engaging”, “non-partisan” and “non-ambiguous”. Finally, Hamilton and Thompson [50] argue that direct experience increases the preference for products that are easy to use compared with those that are more difficult to operate but more desirable.

The diffusion of innovation theory suggests that direct experience involves several features that boost the adoption rate of an innovation [42]. In particular, it increases the trialability and observability of an innovation, thereby reducing any perceived complexity. Moreover, during the decision stage of adopting an innovation, direct experience can be a highly effective means of coping with the inherent uncertainty associated with innovation in general. Demonstrations involving a direct experience are viewed as particularly powerful facilitators of innovation diffusion when conducted in a highly visible manner and when the organisers believe the innovation will be well-received by the public.

### 2.3 The role of general attitudes in the formation of specific attitudes

In addition to direct experience, general attitudes are also important for the formation of specific attitudes. Prislin and Quellette [51, p. 846] conclude that general attitudes are good predictors of specific attitudes, especially “(a) when specific situations are related to the general issue and (b) when the attitudes towards broader objects or issues are evoked before the specific evaluative reactions”. To illustrate this point using a recycling example, Vining and Ebreo [52] argue that generalised concerns for the environment are positively but weakly related to specific attitudes towards recycling. The fact that specific attitudes can be influenced by both direct experience and general attitudes implies that these two aspects need to be analysed simultaneously to calculate their respective net effects.

### 2.4 The goal of the study and research questions

Against this background, the objective of this study is to analyse the effectiveness of promoting LFSHs through a contextually embedded intervention taking a mass campaign of a local utility as a benchmark. The contextually embedded intervention includes an information campaign in a public swimming pool recently equipped with LFSHs, thereby providing an opportunity to experience an LFSH first-hand. In particular, the following three questions are addressed:

- What is the relative effectiveness of the contextualised LFSH intervention compared with the utility’s mass campaign?
- Which specific mechanisms underlie the decision and intention to purchase an LFSH within the contextualised LFSH intervention?
- How important is the role of the positive direct experience with an LFSH as offered at the public swimming pool?

## 3 Methods and materials

### 3.1 Study setting

The utility of the Swiss city of Winterthur (115,492 inhabitants in 2019 [53]) conducted a promotional campaign (hereafter the”utility’s mass campaign”) offering LFSHs for domestic use for 10 Swiss francs instead of 37.70 Swiss francs (approx. €9 instead of €34). The campaign began in November 2016 and ended in April 2017. The only eligibility criterion for subsidised LFSHs was residence in the city. The core element of the campaign was a flyer which included information regarding the benefits associated with LFSHs in terms of cost, water and energy saving, as well as a detachable purchase order form. The flyer was distributed with the energy bills and the quarterly customer magazine to a total of 49,878 [53] households in the city of Winterthur. In addition to flyers, information regarding the campaign was disseminated through various other channels, including local newspapers, the utility’s website, the city of Winterthur’s intranet, the Homeowners’ Association, the Tenants’ Association, an e-newsletter of the utility’s climate fund and the information desk at World Water Day in Winterthur. The campaign could thus be characterised as a mass campaign combining both financial incentive and information-based intervention.

At the same time, the only indoor public swimming pool in the city of Winterthur—hosting around 1,000 visitors per day—was re-opened following a major refurbishment. This included the installation of LFSHs in the communal pre- and post-swim washing areas. The reopening coincided with the beginning of the utility’s mass campaign to promote LFSHs. This quasi-experimental study made use of the simultaneous occurrence of these two initiatives to design and evaluate an intervention that promoted LFSHs in the setting of a swimming pool (hereafter the “contextualized LFSH intervention”).

### 3.2 Contextualised LFSH intervention

The core of the contextualised LFSH intervention was an information campaign targeting visitors to the newly refurbished public swimming pool in the city of Winterthur. They were specifically informed of the following: (1) that they would now be showering using an LFSH, (2) the benefits of LFSHs and (3) the utility’s mass campaign taking place. Informing visitors about new LFSHs was crucial to enable a conscious direct experience since the new showerheads had been designed to be as indistinguishable as possible from conventional showerheads. In addition, the motivation behind the three elements of the campaign was to induce a reflection on and possibly exchanges among visitors regarding their showering experience with a new LFSH, and consequently to increase their willingness to buy a subsidised LFSH for their own households.

**Fig 1:**
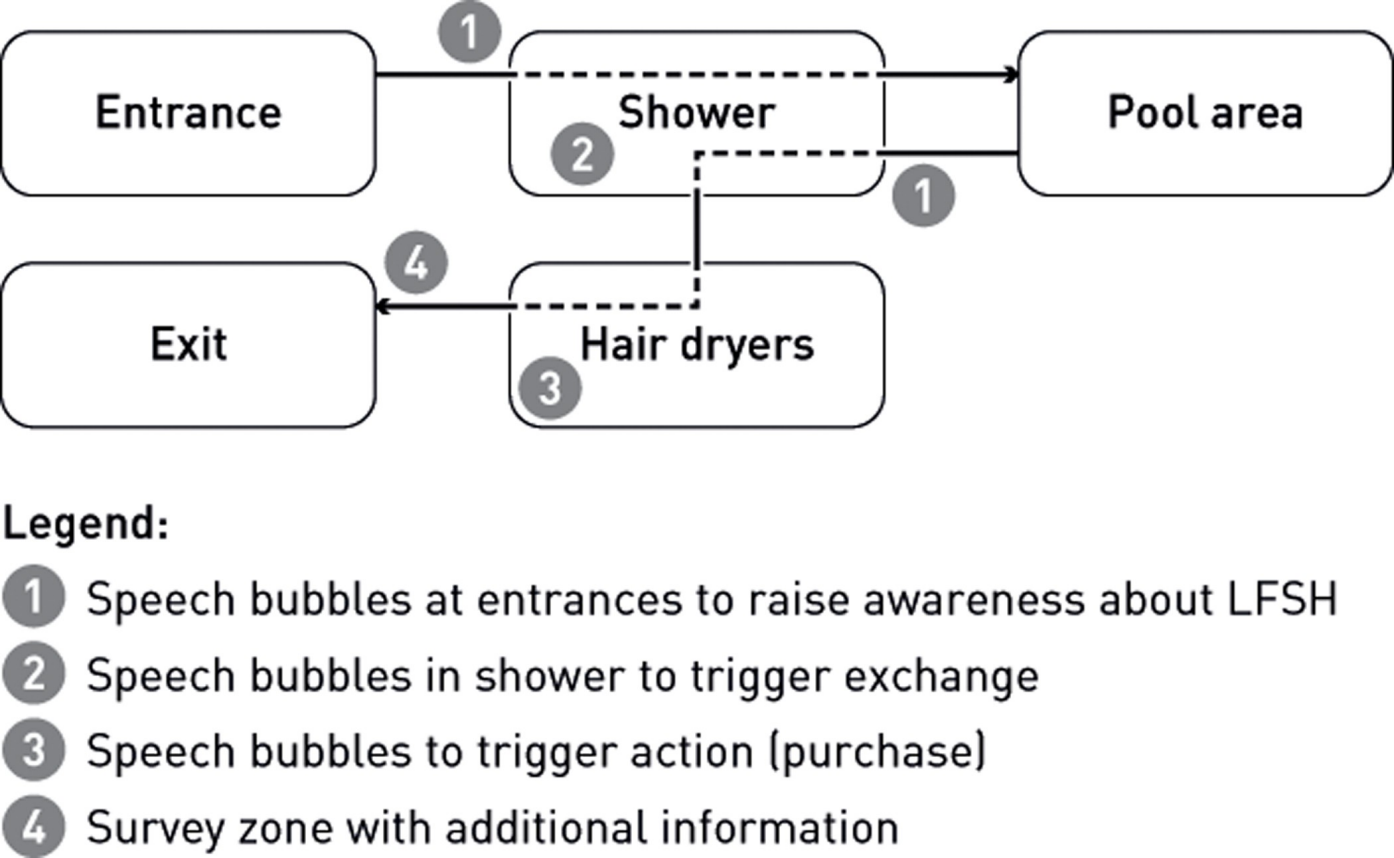
Information campaign in the public swimming pool.

The intervention took place for two weeks in January and February 2017. It was designed with the support of a communication agency and consisted of the following elements (see Fig 1 for an overview):

- Speech bubble stickers at both entrances to the shower rooms, the primary purpose of which was to draw attention to the new LFSHs (see S1 File).
- Speech bubble stickers in the shower rooms, the primary purpose of which was to draw attention to the new LFSHs, to highlight their benefits and to indicate the opportunity to purchase them for home use at a subsidised price during the utility’s mass campaign (see S2 File).
- Speech bubble stickers on the mirrors in the hair-drying area, the primary purpose of which was to draw attention to the survey zone located just a few steps from the exit stairs (see S3 File).
- The survey zone including (a) a table to complete the survey, (b) a poster with more detailed information, particularly regarding the link between warm water and energy consumption, and the saving potential of LFSHs and (c) a flyer stand for promotional material (see S4 File).

### 3.3 Data collection

Data was collected using both an on-site (paper and pencil) and an online survey. Participation in the survey was—of course—voluntary. At the beginning of the survey, participants were informed in writing that their responses would be used for research and teaching purposes only, and analysed and published in an anonymous form. Participants were allowed not to provide any personal details and 156 respondents chose this option. This type of non-invasive research does not require prior approval from an ethics committee according to the Swiss Federal Act on Research Involving Human Beings (HRA).

Up to three team members (consisting of researchers and student assistants) were on-site for several hours per day to recruit participants during periods of high visitor frequency. In total, about 60 hours of on-site presence by survey team members were recorded, and they were positioned in the hair-drying area close to the exit. The survey team was also equipped with a dry LHSH model promoted by the utility’s mass campaign for visitors who were interested in a closer examination. According to the survey team estimate, 40–50% of visitors who passed the survey zone during their presence agreed to take part in the survey.

In addition to the on-site survey, visitors were given the option of completing an online version of the survey. Those interested in participating in the online survey could either read the access information (via a link to the questionnaire website or the respective QR code) on the speech bubble in the hair-drying area or were given the flyer with the corresponding information. This ensured that visitors were able to participate in the survey even if the survey team was not present or if visitors lacked time to complete it spontaneously.

Incentives for participation included a voucher for a free cup of coffee at the swimming pool cafeteria, which was given to each person on completion of the on-site survey. In addition, all respondents were entered into a draw to win a gift voucher worth 100 Swiss francs (85 €) for one of four different online stores (participants could choose). Moreover, to increase participation rates, the coaches of sports clubs and associations that meet regularly at the swimming pool were made aware of the survey by e-mail and asked to motivate their members to take part in the survey. On average, online participation required 10 minutes to complete (median: eight minutes). The time required to complete the on-site version was not precisely tracked, although most participants completed it in 5–15 minutes.

Ultimately, survey participants were matched with purchase orders for LFSHs received by the utility using the names and addresses provided. This allowed us to determine whether a respondent took advantage of the utility’s mass campaign, and, if so, whether this happened before or after the contextualised LFSH intervention at the swimming pool.

### 3.4 Survey structure and variables

*The quality of direct experience with an LFSH* (see S1 Table for question-wording and descriptive statistics) measured participant satisfaction with new LFSHs in respect of (1) water pressure, (2) water quantity and (3) overall experience. The items were self-constructed and measured using a five-point Likert scale labelled from “do not agree at all” (1) to “completely agree” (5). Owing to the low value of Cronbach’s alpha (α = .31), the items were considered separately in the analysis rather than aggregating them to a quality of direct experience scale.

*Social interaction* (see S1 Table for question-wording and descriptive statistics) referred to the intensity of social interaction regarding new LFSHs differentiating between (1) conversations with known persons and (2) conversations with unknown persons. The items were self-constructed and measured on a five-point Likert scale labelled from “does not apply” (1) to “long, in-depth conversation” (5). The sum of these two items was taken as an aggregate measure of social interaction. Hence, the scale of the aggregate measure was 1–10. In addition, participants were asked to describe the content of their discussions briefly using an open-ended question.

*Attitudes towards LFSHs* (see S1 Table for question-wording and descriptive statistics) included (1) impact on potable water, (2) environment protection, (3) comfort, (4) influence on shower duration and (5) whether participants generally believed that LFSHs were suitable for them or considered positive in general. The items were formulated based on the guidelines provided by [54], adapted to the context of LFSHs and measured using a five-point Likert scale labelled from “do not agree at all” (1) to “completely agree” (5). A scale of attitudes towards LFSHs (Cronbach’s α = .84) was developed by taking the mean value of these items.

*General attitudes towards saving warm water* (see S1 Table for question-wording and descriptive statistics) included (1) an understanding of the warm water-energy nexus, (2) attitudes towards saving warm water through the deployment of new technologies, (3) attitudes towards saving warm water through behavioural changes, (4) attitudes towards wasting water by excessive showering practices and (5) attitudes regarding whether more could be done to save warm water. The items were self-constructed and measured using a five-point Likert scale labelled from “do not agree at all” (1) to “completely agree” (5). Owing to the low value of Cronbach’s alpha (α = .38), the items were considered separately in the analysis rather than aggregating them to a general attitudes scale.

*Subjective norm* (see S1 Table for question-wording and descriptive statistics) included questions to determine how participants perceived (1) the expectations and (2) approval of their peers regarding participants’ showering and warm-water-saving behaviour as well as how participants perceived (3) the showering and (4) the warm-water-saving behaviour of their peers. The items were formulated based on the guidelines provided by [54] and measured using a five-point Likert scale labelled from “do not agree at all”(1) to “completely agree” (5). The mean of these four items was used to develop a subjective norm scale (α = .85).

*Perceived behavioural control* (see S1 Table for question-wording and descriptive statistics) included questions regarding (1) whether participants believed LFSH installation was complicated, (2) whether participants knew how to install an LFSH and (3) whether participants had been involved in decision-making regarding the purchase of an LFSH for their household. The items were formulated based on the guidelines provided by [54] and measured using a five-point Likert scale labelled from “do not agree at all” (1) to “completely agree” (5). The mean of these three items (after recoding the two negatively formulated items) was used to develop a scale of perceived behavioural control (α = .58).

*Intention to purchase an LFSH* (see S1 Table for question-wording and descriptive statistics) was used to measure the intention of the participants to purchase an LFSH during the utility’s mass campaign. The question was formulated based on the guidelines provided by [54] and measured using a five-point Likert scale labelled from “do not agree at all” (1) to “completely agree” (5).

For the question related to *previous experience with an LFSH* (see S2 Table for question-wording and descriptive statistics), the participants could answer (1) that they already used an LFSH at home, (2) that they already had experience of an LFSH, (3) that they had no experience of an LFSH or (4) that they did not know whether or not they had experience of an LFSH. Based on this question, the variable “direct experience at home (DEH)” was constructed by assigning 1 to those who indicated that they already used an LFSH at home and 0 otherwise.

Finally, *socio-demographic data* for the participants were collected. Participants were also asked to provide addresses to allow the determination of survey participants who took advantage of the utility’s mass campaign prices.

### 3.5 Sample description

Overall, 402 from a total of 412 people provided useable responses (282 of 283 on-site and 120 of 129 online). Around 48% were women (Winterthur: 50.8% [53]), and 52% were men (Winterthur: 49.2% [53]). The mean age was approximately 42 years (Winterthur: 40.7 [53]). Regarding the level of education, 41.7% reported having at least a university degree (44.8% greater region Zurich [55]). Hence, our sample can be considered as largely representative for the population of Winterthur regarding gender, age and level of education.

As indicated in Table 1, of the 298 participants who provided their addresses, 228 were residents of the city of Winterthur and 70 were residents of other municipalities. A total of 104 people did not provide an address in the questionnaire. Moreover, 189 participants reported that they already owned an LFSH (not necessarily purchased as part of the utility’s mass campaign) compared with 193 participants who reported not owning one. The remaining 20 participants did not answer this question.

**Table 1:** Place of residence and possession of an LFSH before the contextualised LFSH intervention.

Place of residence		Possession of an LFSH before the survey	Yes	No	No indication	Total
Winterthur			109	113	6	228 (57%)
Another municipality			35	31	4	70 (17%)
No address given			45	49	10	104 (26%)
Total			189 (47%)	193 (48%)	20 (5%)	402 (100%)

### 3.6 Data analysis

The data were analysed in four consecutive steps. Firstly, we conducted a simple comparison of purchase order ratios between the utility’s mass campaign and the contextualised LFSH intervention. Secondly, we explored differences in purchase intentions and perceived behavioural control between purchasers and non-purchasers using t-tests. Thirdly, we conducted a linear regression to explain the intention to purchase a subsidised LFSH based on the TPB. Fourthly, we explored how general attitudes towards saving warm water, direct experience at the swimming pool and direct experience at home related to attitudes towards LFSHs by comparing the regression coefficients of a linear regression analysis. The final step also compared the adjusted R^2^ of the baseline model including all three blocks of independent variables (attitudes towards saving warm water, positive direct experience at the swimming pool and direct experience at home) with the adjusted R^2^ of the following models:

- baseline model minus attitudes towards saving warm water,
- baseline model minus positive direct experience at the swimming pool and
- baseline model minus direct experience at home.

Comparison of the purchase order ratios (1) and analysis of the differences between purchasers and non-purchasers (2) focuses on the residents of the city of Winterthur who had not taken advantage of the utility’s mass campaign before the contextualised LFSH intervention and provided their addresses in the on-site or online survey of the contextualised LFSH intervention (N = 206). Our focus on residents of Winterthur was required to compare the effect of the utility’s mass campaign (which only covered residents of the city) with the contextualised LFSH intervention. On the other hand, analysis of the intention to purchase an LFSH (3) and analysis of attitudes towards LFSHs (4) had a more general focus and, consequently, included the entire sample (N = 402).

## 4 Results

### 4.1 Effectiveness of the contextualised LFSH intervention vs. the utility’s mass campaign

As shown in Table 2, 34 participants (32 households) in the survey took advantage of the utility’s mass campaign following the contextualised LFSH intervention. This corresponds to a purchase order ratio of 16.5% in terms of individual participants and 16.8% for households. Hence, the purchase order ratio of the combined intervention—contextualised LFSH intervention and utility mass campaign—was almost twice as high as the purchase order ratio associated with the utility’s mass campaign alone (8.9%).

**Table 2:** Purchase status of participants living in Winterthur.

Purchase status	Contextualised LFSH intervention (individuals)^a)^	Contextualised LFSH intervention (households)^b)^	Utility’s mass campaign (households)
Purchase after intervention	34	32	4,400^c)^
Total	206^d)^	190	49,688^e)^
**Purchase order ratio**	**16.5%**	**16.8%**	**8.9%**

a) According to the matches between addresses with purchase orders received by the utility

b) Only one person per address counted.

c) See [56].

d) After excluding 22 participants who purchased an LFSH before the contextualised LFSH intervention.

e) The total of 49,878 households from Winterthur [53] minus 190 households living in Winterthur, which were surveyed during the contextualised LFSH intervention and did not take advantage of the utility’s mass campaign before the contextualised LFSH intervention.

### 4.2 Determinants of an LFSH purchase

In line with the TPB [19], participants from Winterthur who purchased a subsidised LFSH after the contextualised LFSH intervention reported a significantly higher purchase intention (*M* = 4.65, *SD* = .80) compared with those from Winterthur who did not purchase an LFSH after the contextualised LFSH intervention (*M* = 3.23, *SD* = 1.20): *t*(53) = 7.47, *p* < .001, *r* = .72 (independent t-test, two-tailed). For perceived behavioural control, significant but smaller differences were found between purchasers (*M =* 4.25, *SD* = .84) and non-purchasers (*M* = 3.71, *SD* = 1.01), *t*(200) = 2.97, *p* < .010, *r* = .20 (independent t-test, two-tailed).

The results of the linear regression presented in Table 3 suggest that only attitudes towards LFSHs and perceived behavioural control were significantly related to the intention to purchase an LFSH. Thereby, the effect of the attitudes towards LFSHs was substantially stronger than the effect of perceived behavioural control. The subjective norm was not significantly related to the intention to purchase an LFSH. These results do not change if those who had an LFSH before the contextualised LFSH intervention (according to their statement in the survey) are not considered in the regression.

**Table 3:** Linear regression to explain the intention to purchase an LFSH.

Independent variable		B	SE	β
	(Constant)	-1.418	.533	
	Attitudes towards LFSHs	.755	.115	.410***
	Perceived behavioural control	.249	.077	.185**
	Subjective norm	.166	.093	.112

Dependent variable: “I intend to take advantage of the utility’s promotion campaign (LFSH for 10.- instead of 37.70.-)”.

N = 233, Adj. R^2^ = .260, F = 26.768

**p<0.01,

***p<0.001

Social interaction at the swimming pool regarding the new LFSHs was very low (*M* = 2.9, *SD* = 1.47) and does not correlate with the subjective norm: *r* = .067, *p* = 0.2, (two-tailed). However, social interaction does positively correlate with the item “water pressure was too low for me”: *r* = .167, *p* = 0.001 (two-tailed). Analysis of the answers to the open-ended question regarding the content of discussions showed that those who complained about the showering experience had higher means for the social interaction scale compared with those who positively discussed their showering experience (see S1 Fig for details).

### 4.3 The role of direct experience at the swimming pool in the formation of attitudes towards LFSHs

As shown in Table 3, attitudes towards LFSHs had a strong and significant effect on the intention to purchase an LFSH. In this section, we analyse the relation of (1) direct experience of an LFSH (provided by the contextualised LFSH intervention at the swimming pool), (2) direct experience at home of a personal LFSH and (3) general attitudes towards saving warm water (hereafter referred to as “general attitudes”) to the attitudes towards LFSHs.

In the baseline model (Model 1) of the linear regression analysis, all the above-mentioned independent variables were included—general attitudes, direct experience at the swimming pool and direct experience at home. In Models 2–4, we analyse how the share of explained variance changed after excluding general attitudes (Model 2), direct experience in the swimming pool (Model 3) and direct experience at home (Model 4).

Table 4 shows that in the baseline model (Model 1), four out of five general attitude items, two out of three items concerning direct experience at the swimming pool and the variable capturing direct experience at home had significant effects on attitudes towards LFSHs. This implies that all three independent variables (general attitudes, direct experience at the swimming pool and direct experience at home) are significantly related to attitudes towards LFSHs. Positive general attitudes and a positive direct experience are associated with a positive effect, while negative general attitudes and a negative general experience are associated with a negative effect on attitudes towards LFSHs. In addition, having direct experience of an LFSH at home was associated with a negative effect on attitudes towards LFSHs.

**Table 4:** Regression analysis to explain attitudes towards LFSHs.

Independent variables		Model 1 adjR^2^ = .48	Model 2 adjR^2^ = .16	Model 3 adjR^2^ = .43	Model 4 adjR^2^ = .45
(Const.)	B	1.542	4.243	1.655	1.168
	SE	.259	.182	.252	.248
GA1: A lot of energy can be saved by saving warm water.	B	.079		.089	.079
	SE	.040		.041	.041
	β	.088*		.098*	.087
GA2: It is important to me that new technologies are introduced to save warm water.	B	.338		.360	.346
	SE	.046		.047	.047
	β	.339***		.362***	.347***
GA3: You cannot request people to change their showering behaviour to save warm water.	B	-.008		-.019	-.012
	SE	.022		.022	.023
	β	-.015		-.035	-.021
GA4: It is unacceptable that a valuable resource such as water is wasted by showering.	B	.114		.118	.115
	SE	.032		.033	.033
	β	.171***		.177***	.172**
GA5: In my opinion, more could be done to reduce warm water consumption.	B	.112		.130	.116
	SE	.037		.038	.038
	β	.149**		.172**	.154**
DESP1: The water pressure was too low for me.	B	-.045	-.081		-.046
	SE	.020	.024		.020
	β	-.090*	-.162**		-.091*
DESP2: The water quantity was fine for me.	B	.095	.150		.097
	SE	.038	.047		.039
	β	.159*	.251**		.162*
DESP3: Generally, it was a pleasant showering experience.	B	.008	.030		.005
	SE	.039	.049		.040
	β	.013	.048		.009
DEH: Previous experience of an LFSH.	B	-.223	-.284	-.223	
	SE	.055	.069	.056	
	β	-.156***	-.199***	-.156***	

GA = General attitudes towards saving warm water (scale from 1–5)

DESP = Direct experience of an LFSH

DEH = Have you had any experience of a low-flow showerhead before today’s visit to Geiselweid swimming pool? 1: Yes, I have one at home; 0: Yes, away from home/No/I don’t know

Dependent variable = attitudes towards LFSHs (a scale)

N = 366, adjR^2^ = .468 for step 1, ∆adjR^2^(Step 2) = -.310***, ∆adjR^2^(Step 3) = -.034***, ∆adjR^2^(Step 4) = -.023***

*p<0.05,

**p<0.01,

***p<0.001

Regarding the relative importance of the three independent variables, the adjusted R^2^ decreased by 31% when general attitudes were excluded from the baseline model, whereas excluding direct experience at the swimming pool from the baseline model was associated with a 3.4% decrease and excluding direct experience at home with a 2.3% decrease of the adjusted R^2^ (see footnote Table 4). These changes in the adjusted R^2^ are significant. This implies that general attitudes explain a large proportion of the variance in attitudes towards LFSHs. The share of variance explained by direct experience (at the swimming pool and at home) was considerably lower but still significant. Direct experience at the swimming pool explained slightly more variance in attitudes towards LFSH than direct experience at home.

## 5 Discussion

### 5.1 Summary and discussion of findings

Comparisons of purchase order ratios showed that the contextualised LFSH intervention combined with the utility’s mass campaign was almost twice as effective as the utility’s mass campaign alone.

During the contextualised LFSH intervention, purchasers of a subsidised LFSH were associated with a significantly higher intention to purchase an LFSH and a significantly higher perceived behavioural control (the two TPB behavioural predictors) than the non-purchasers. Thereby, the difference regarding the intention to purchase an LFSH was higher than the difference regarding behavioural control. In our study, we have taken perceived behavioural control to mean the ability to install an LFSH rather than the ability to use one. Since none of the elements in the contextualised LFSH intervention addresses this notion of perceived behavioural control, we have not discussed its effect in greater detail here.

Attitudes towards LFSHs were the strongest predictor of the intention to purchase an LFSH followed by perceived behavioural control. The subjective norm was neither correlated with social interactions regarding LFSHs at the swimming pool nor associated with a significant effect on the intention to purchase an LFSH.

One crucial feature offered in the swimming pool context was the direct experience of an LFSH. While a positive direct experience was positively related to attitudes towards LFSHs, a negative direct experience was negatively related to them. In addition to a positive direct experience, attitudes towards LFSHs were positively related to positive general attitudes towards saving warm water and were negatively influenced by direct experience at home. These findings clearly suggest that the generally positive attitudes towards LFSHs among the respondents were partly strengthened by the positive direct experience of an LFSH at the swimming pool. In addition, a positive direct experience of an LFSH at the swimming pool contrasted with the negative experience of an LFSH of most domestic users. Finally, attitudes towards LFSHs contributed substantially to a greater intention to purchase a subsidised LFSH, which in turn was significantly higher among the LFSH purchasers than among non-purchasers.

Interestingly, the subjective norm had neither a significant effect on the intention to purchase a subsidised LFSH, nor was it influenced by social interactions at the swimming pool. Based on the data, it can be concluded that in contrast to products such as solar panels [39], social comparison and social influence processes are probably less effective in promoting products with less visibility and distinctive power, such as LFSHs. This is in line with Rogers [42], who argues that the rate of the adoption of an innovation depends on the relative advantage over its predecessor (among other factors). Rogers [42] also suggests that the relative advantage of an innovative product is based on its status-conferring nature in the case of highly visible (to others) innovations, whereas this is not true for less visible (to others) innovations, such as domestic LFSHs.

The low level of recorded social interaction related to LFSHs suggests that it is difficult to stimulate discussions about them. Moreover, the few who did discuss LFSHs used the opportunity to complain about them rather than to praise them—although the average level of satisfaction with the LFSHs among the respondents was reasonably high. This suggests that the social interaction effect is difficult to control and can lead to unintended outcomes.

### 5.2 Critical reflections

A considerable methodological asset of this study was its real-world setting. Respondents were not provided with hypothetical contexts but exposed to authentic choices and experiences. Consequently, this setting contributes to a high external validity of the results. Studies on sustainability solutions with participatory control over interventions, such as the contextualised LFSH intervention, provide valuable, actionable, evidence-based knowledge [57]; however, such studies are relatively recent and still require established design and evaluation methods [57–59]. Hence, this study enriches this relatively young body of literature with new insights regarding possible designs and evaluation methods for studies on sustainability solutions with participatory control over interventions. Nevertheless, the high external validity of the results associated with the real-world setting of this study involved a specific compromise regarding the internal validity of the results. For example, the setting does not exclude the possibility of self-selection bias—the possibility that the higher purchase order ratio, higher purchase intention or more favourable attitudes toward LFSHs in the sample were not necessarily due to the contextualised LFSH intervention but to sample characteristics, such as more favourable environmental and energy-saving attitudes in general.

When interpreting the results, it is important to keep in mind the quasi-experimental design of this study. In particular, it should be noted that a comparison of the purchase order ratios of the combined intervention—contextualised LFSH intervention plus the utility’s mass campaign—and the utility’s mass campaign alone should be taken only as a very broad indicator of the relative effectiveness of the two approaches. Comparison of the purchase order ratios should not be interpreted as a treatment effect in the sense of a randomised control trial. Assignment to the group that was exposed to the contextualised LFSH intervention and the unexposed group was neither controlled nor random, so different targeting and comparability issues should be kept in mind when interpreting our results. Firstly, the contextualised LFSH intervention was launched two months after the launch of the utility’s mass campaign, suggesting that many early adopters had already purchased a subsidised LFSH before the start of the contextualised LFSH campaign. This implies that the purchase order ratio associated with the contextualised LFSH intervention should be interpreted rather conservatively. Secondly, the people who were exposed to the contextualised LFSH intervention but did not take part in the survey were unaccounted for in the purchase ratio of the contextualised LFSH intervention. This might artificially inflate the purchase order ratio of the contextualised LFSH intervention. Thirdly, our design does not allow us to test for cross-contamination between the groups—people who were exposed to the contextualised LFSH intervention could have discussed their experience of the new LFSHs in the swimming pool with others, such as those they live with. This is another factor that would tend to reduce the estimated effect of contextualised LFSH intervention. While several issues arise from the lack of control, lack of targeting precision and lack of randomisation, these act in different directions and may partly negate each other.

### 5.3 Further research

Interventions to promote LFSHs would be regarded as even more effective if they led directly to other energy-saving behaviours (positive spill-over). At the same time, however, such interventions could also lead to increased warm water use as a consequence of an energy efficiency improvement perception (rebound effect). From this perspective, an interesting avenue for further research would be to investigate the extent to which the purchase of an LFSH is susceptible to positive spill-over or rebound effect [60,61]. Since showering, in particular, can be considered a highly habitual behaviour [62], it would be interesting to analyse the long-term consequences of adopting LFSHs at home. Moreover, our finding that a substantial proportion of respondents already have an LFSH at home suggests that designing and evaluating a campaign with the focus on descriptive social norms would also be a useful exercise.

## 6 Conclusion

Promoting LFSHs in the contextually proximate setting of a public swimming pool allowed visitors to experience an LFSH without any effort on their part while pursuing a leisure activity. According to our findings, this is a promising strategy for promoting this technology. Such an approach offers a pleasing compromise between the “one-size-fits-all” and tailored intervention approaches. On the one hand, it involves the target group with a wide range of socio-demographic characteristics. On the other hand, this target group is presumably associated with a more conscious approach to showering and warm water consumtion than the broader population owing to its leisure orientation.

On a very general level, this study shows that energy efficiency refurbishments for public buildings, such as the installation of LFSHs in a public swimming pool, have a role model potential that goes beyond improvements in energy efficiency. Where an energy-saving device is also available for use in the home, these trial arenas allow visitors to test and directly experience the innovation, confront any prejudices and update possible previous experiences. To achieve this, refurbishments need to be accompanied by information campaigns and possibly public events because the energy efficiency characteristics of a refurbishment are rarely self-evident, as illustrated by the example of LFSHs.

## Supporting information

S1 Fig (PPTX)

S1 Table Overview of the items and variables from the survey. (DOCX)

S2 Table Previous experience with an LFSH. (DOCX)

S1 File (DOCX)

S2 File (DOCX)

S3 File (DOCX)

S4 File (DOCX)
