# Supplementary material for: Exploring the role of positive direct experience in the adoption of energy efficient technologies: evidence from a Swiss field study on the promotion of low-flow showerheads
Source: PLoS One. 2020 Mar 16;15(3):e0230255. doi: 10.1371/journal.pone.0230255 (PMC7075542; doi:10.1371/journal.pone.0230255)
Supplement: S1 Fig — (PPTX) [file pone.0230255.s001.pptx]

## Slide 1
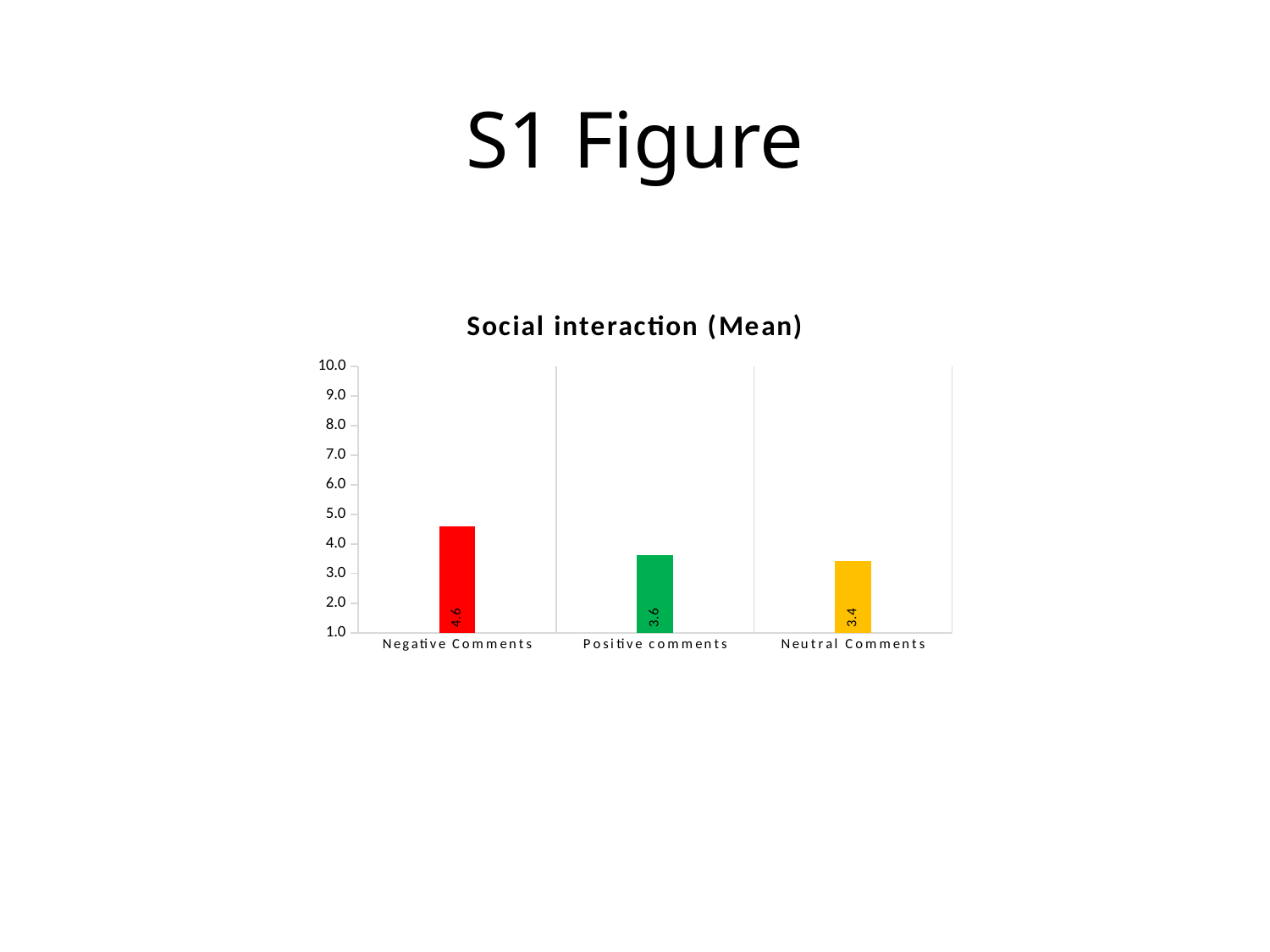

# S1 Figure
### Chart: Social interaction (Mean)
| Category | Gespräch_Sum |
|---|---|
| Negative Comments | 4.592592592592593 |
| Positive comments | 3.625 |
| Neutral Comments | 3.4324324324324325 |
