# Supplementary material for: Exploring the role of positive direct experience in the adoption of energy efficient technologies: evidence from a Swiss field study on the promotion of low-flow showerheads
Source: PLoS One. 2020 Mar 16;15(3):e0230255. doi: 10.1371/journal.pone.0230255 (PMC7075542; doi:10.1371/journal.pone.0230255)
Supplement: S1 Table — (DOCX) [file pone.0230255.s002.docx]

S1 Table: Overview of the items and variables from the survey

| Variable | Items and scales (bold) | | N | M | SD |
| --- | --- | --- | --- | --- | --- |
| Direct experience with a LFSH | The water pressure was too low for me (negative). | | 393 | 2.23 | 1.45 |
|  | I was satisfied with the amount of water. | | 397 | 4.12 | 1.19 |
|  | Overall, I found the shower feeling pleasant. | | 397 | 4.18 | 1.16 |
| Social interaction | I had a conversation about the low-flow showerheads with somebody I know. | | 389 | 1.69 | 1.01 |
|  | I had a conversion about the low-flow showerheads with an unknown person. | | 383 | 1.29 | .71 |
|  | **Scale: Social interaction^a)^** | | **392** | **2.9** | **1.47** |
| Attitudes towards LFSHs | The use of a water-saving showerheads suits me. | | 392 | 4.38 | .95 |
|  | A water-saving showerhead contributes to drinking water protection. | | 388 | 4.33 | 1.04 |
|  | Water-saving showerheads are comfortable. | | 385 | 3.93 | 1.06 |
|  | Water-saving showerheads contribute to environmental protection. | | 386 | 4.60 | .79 |
|  | Overall, water-saving showerheads are a good thing. | | 390 | 4.66 | .73 |
|  | **Scale: Attitudes towards LFSHs^b)^** | | **392** | **4.4** | **.73** |
| General attitudes towards saving warm water | A lot of energy can be saved by saving warm water. | | 390 | 4.53 | .79 |
|  | It is important to me that new technologies are introduced to save warm water. | | 392 | 4.62 | .73 |
|  | You cannot request that people change their showering behaviour to save warm water. | | 387 | 2.12 | 1.31 |
|  | It is unacceptable that a valuable resource such as water is wasted by showering. | | 388 | 4.12 | 1.06 |
|  | In my opinion, more could be done to reduce warm water consumption. | | 389 | 4.22 | .97 |
| Subjective norm | People who are important to me expect me to save warm water. | | 381 | 3.10 | 1.31 |
|  | People who are important to me like it when I save warm water. | | 380 | 3.79 | 1.13 |
|  | People who are important to me save warm water. | | 379 | 3.40 | .982 |
|  | People who I care about shower in a water-saving way. | | 381 | 3.19 | 1.05 |
|  | **Scale: Subjective norm^d)^** |  | **384** | **3.4** | **.95** |
| Perceived behavioural control | The installation of a water-saving showerhead is complicated (negative). | | 377 | 1.97 | 1.10 |
|  | I know how to install a low-flow showerhead. | | 377 | 3.76 | 1.41 |
|  | The decision to install a water-saving showerhead is made by someone else (negative). | | 375 | 2.17 | 1.44 |
|  | **Scale: Perceived behavioural control^c)^** | | **381** | **3.9** | **.98** |
| Intention to purchase a LFSH | I intend to take advantage of the promotion campaign (water-saving showerhead for 10.- instead of 37.70.-). |  | 239 | 3.26 | 1.33 |

Notes. All scales range from 1 to 5. For social interaction, scale points are labelled 1 = ‘does not apply’ and 5 = ‘long, intense conversation’. For all other items, 1 = ‘do not agree at all’ and 5 = ‘completely agree’. Original language: German.

a) As a measure of the level of social interaction, the sum of the following two items was taken: (1) ‘I had a conversation about the low-flow showerheads with somebody I know’; (2) ‘I had a conversion about the low-flow showerheads with an unknown person’.

b) Variable attitudes towards LFSHs were calculated as the mean value of the following five items (Cronbach’s α=.84): (1) ‘The use of a water-saving showerheads suits me’; (2) ‘A water-saving showerhead contributes to drinking water protection’; (3) ‘Water-saving showerheads are comfortable’; (4) ‘Water-saving showerheads contribute to environmental protection’; (5) ‘Overall, water-saving showerheads are a good thing’.

c) The variable *perceived behavioural control* was calculated as the mean value of the following three items (α=.58): (1) ‘The installation of a water-saving showerhead is complicated (negative)’; (2) ‘The installation of a water-saving showerhead is complicated (negative)’; (3) ‘The decision to install a water-saving showerhead is made by someone else (negative)’.

d) The variable *subjective norm* was calculated as the mean value of the following four items (α=.85): (1) ‘People who are important to me expect me to save warm water’; (2) ‘People who are important to me like it when I save warm water’; (3) ‘People who are important to me save warm water’; (4) ‘People who I care about shower in a water-saving way’.
