# Supplementary material for: Exploring the role of positive direct experience in the adoption of energy efficient technologies: evidence from a Swiss field study on the promotion of low-flow showerheads
Source: PLoS One. 2020 Mar 16;15(3):e0230255. doi: 10.1371/journal.pone.0230255 (PMC7075542; doi:10.1371/journal.pone.0230255)
Supplement: S2 Table — (DOCX) [file pone.0230255.s003.docx]

S2 Table: Previous experience with a LFSH.

| Category | Freq. | % |
| --- | --- | --- |
| Yes, I have one at home | 189 | 49.5 |
| Yes, away from home (holiday, public place, etc.) | 76 | 19.9 |
| No | 80 | 20.9 |
| I don’t know | 37 | 9.7 |
| Total | 382 | 100.0 |

Item: Have you already had any experiences with a low-flow showerhead before today’s stay at Geiselweid swimming pool?

Note: Original language: German.
