# Supplementary figures and images for: Exploring the role of positive direct experience in the adoption of energy efficient technologies: evidence from a Swiss field study on the promotion of low-flow showerheads
Source: PLoS One. 2020 Mar 16;15(3):e0230255. doi: 10.1371/journal.pone.0230255 (PMC7075542; doi:10.1371/journal.pone.0230255)

Online Resource 1: Speech bubble sticker on the both entrances of the shower room


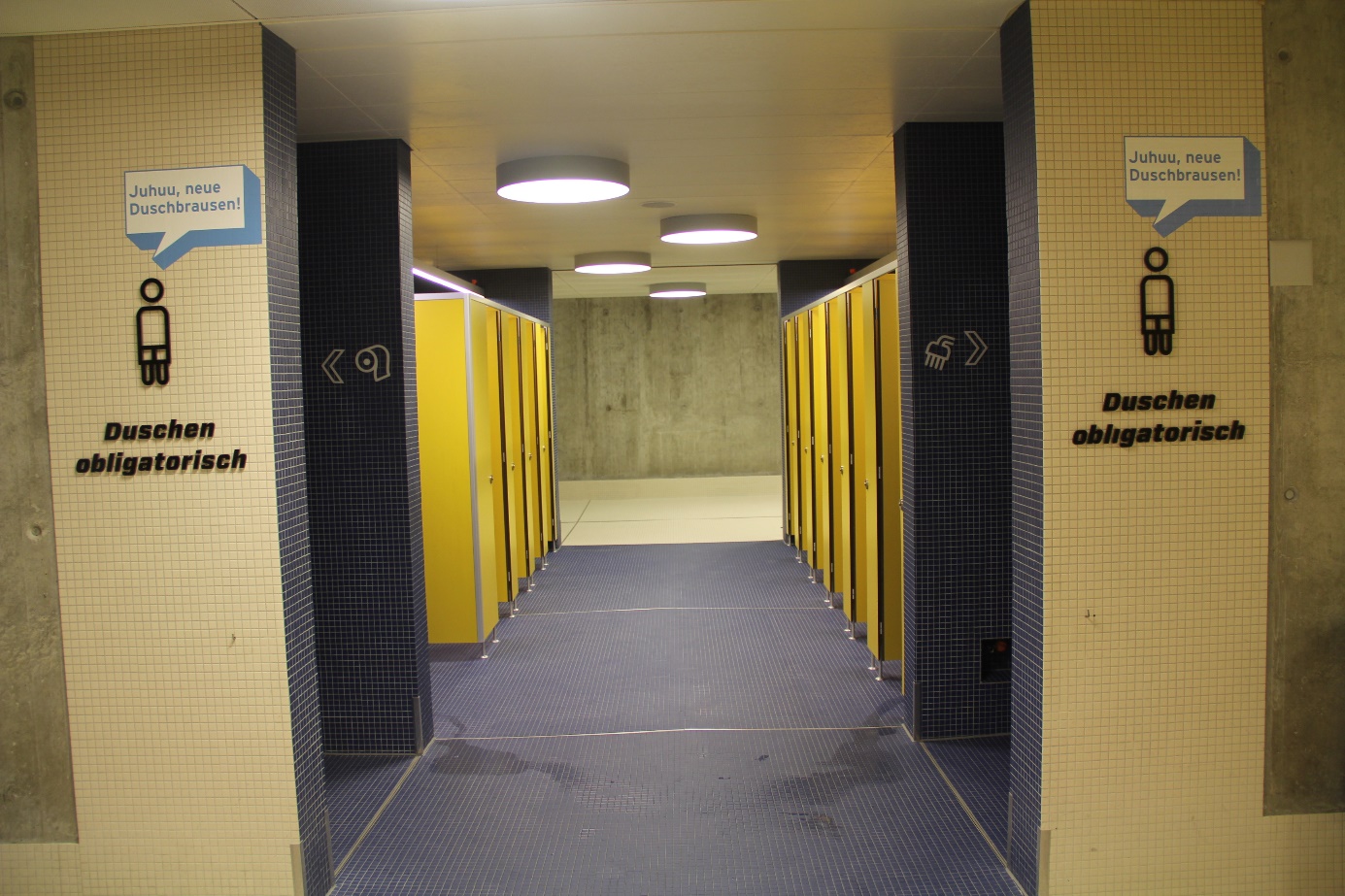

Supplement: S1 File — (DOCX) [file pone.0230255.s004.docx]

Online Resource 2: Speech bubble stickers in the group and individual shower rooms

| 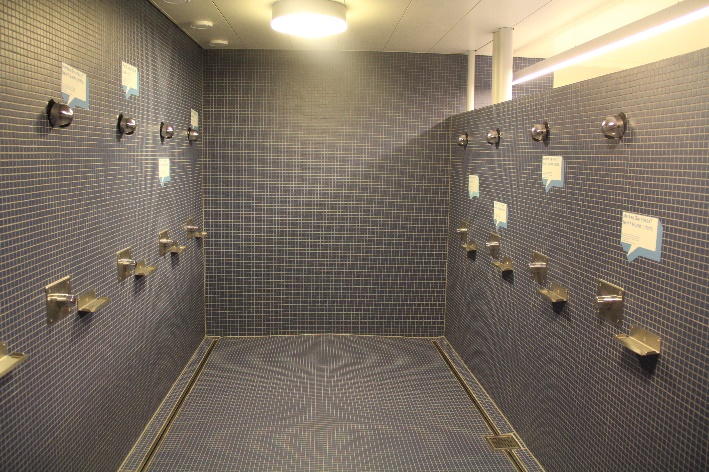 | 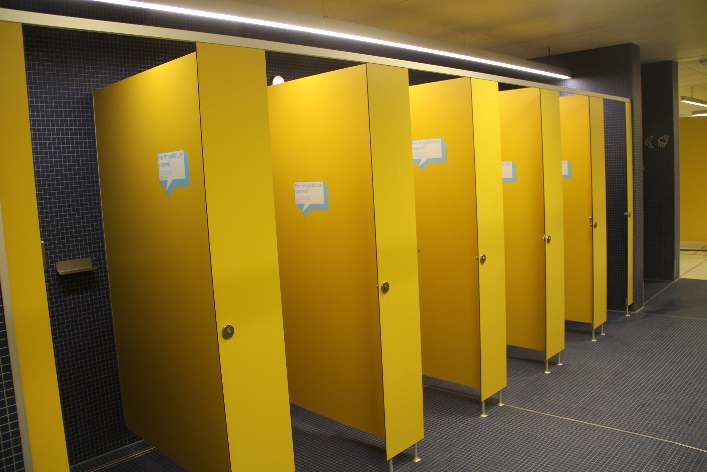 |
| --- | --- |

Supplement: S2 File — (DOCX) [file pone.0230255.s005.docx]

Online Resource 3: Speech bubble stickers on the mirrors in the hair drying area


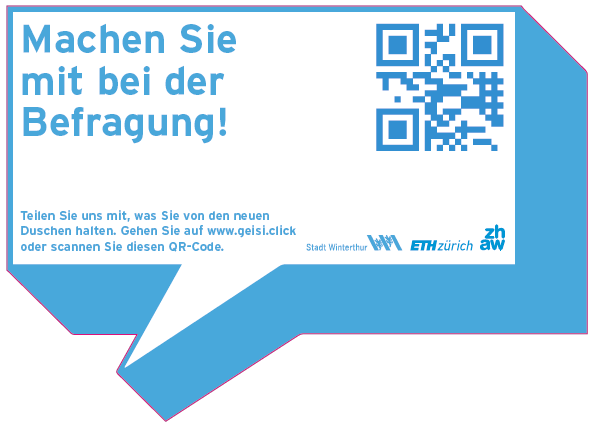


| 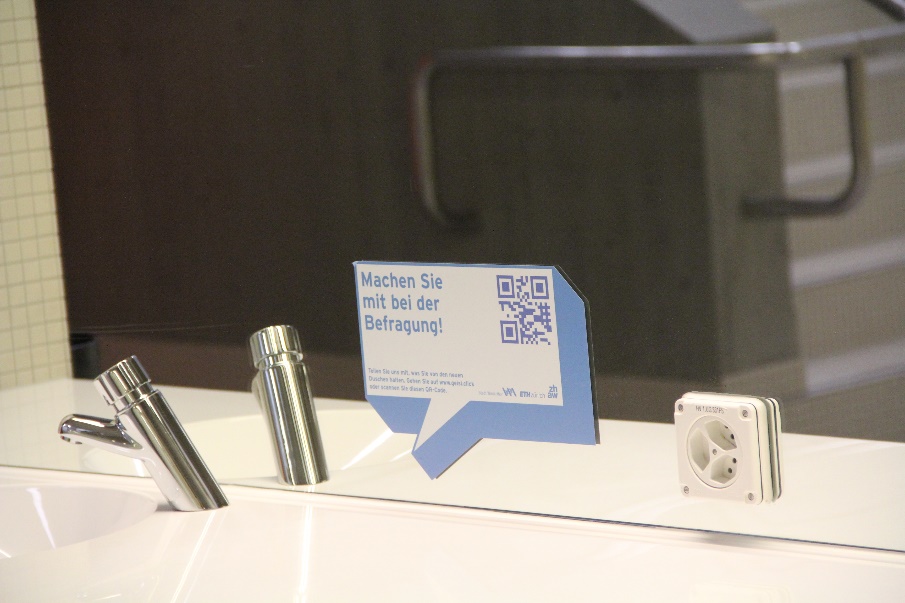 | 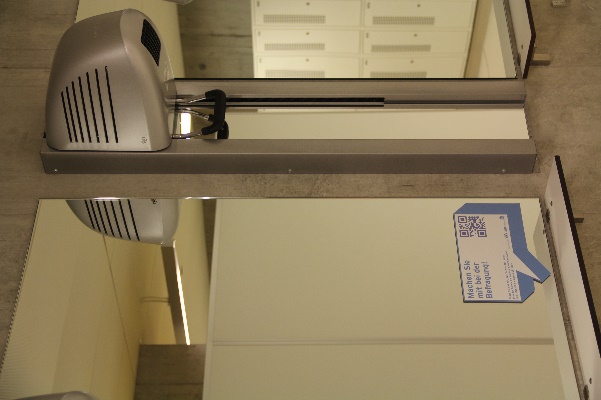 |
| --- | --- |

Supplement: S3 File — (DOCX) [file pone.0230255.s006.docx]

Online Resource 4: Survey zone

| 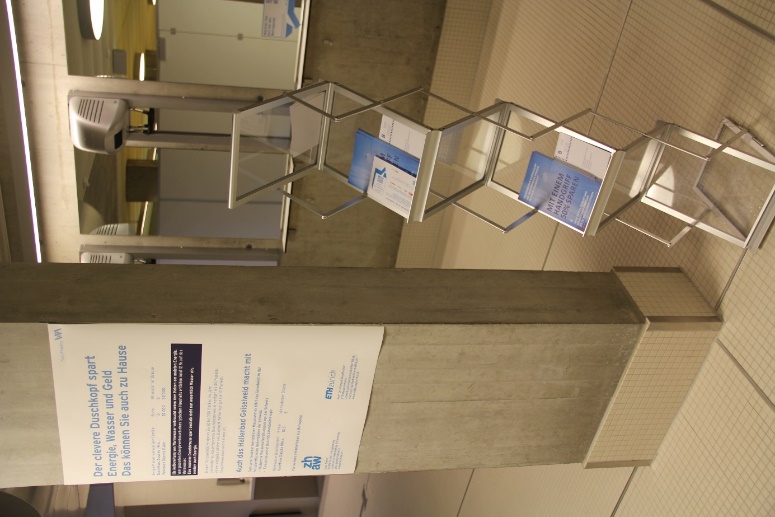 | 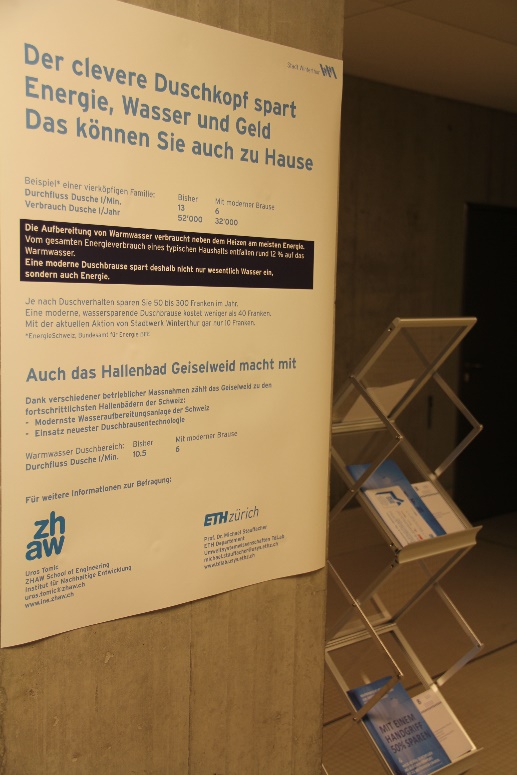 |
| --- | --- |

Supplement: S4 File — (DOCX) [file pone.0230255.s007.docx]
